# Tuberculosis among Healthcare Workers in Southeastern China: A Retrospective Study of 7-Year Surveillance Data

**DOI:** 10.3390/ijerph111112042

**Published:** 2014-11-20

**Authors:** Bin Chen, Xiaomeng Wang, Jiemin Zhong, Songhua Chen, Beibei Wu, Hui-Chi Yeh, Zhenggang Jiang, Zhengting Wang, Hua Gu, Jianmin Jiang

**Affiliations:** 1 Department of Science Research Management, Zhejiang Provincial Center for Disease Control and Prevention, Hangzhou 310051, China; E-Mails: bchen@cdc.zj.cn (B.C.); zhgjiang@cdc.zj.cn (Z.J.); ztwang@cdc.zj.cn (Z.W.); 2 Department of Tuberculosis Control and Prevention, Zhejiang Provincial Center for Disease Control and Prevention, Hangzhou 310051, China; E-Mails: xmwang@cdc.zj.cn (X.W.); jmzhong@cdc.zj.cn (J.Z.); shchen@cdc.zj.cn (S.C.); bbwu@cdc.zj.cn (B.W.); 3 Politics & International Relations, Social Sciences, University of Southampton, Southampton SO17 1BJ, UK; E-Mail: H.Yeh@soton.ac.uk

**Keywords:** tuberculosis (TB), healthcare workers (HCWs), surveillance

## Abstract

The baseline prevalence and characteristics of tuberculosis (TB) among general healthcare workers (HCWs) in southeastern China remains unknown. We conducted a retrospective study based on the TB surveillance data in Zhejiang Province from 2005 to 2011, which were extracted from the national Tuberculosis Information Management System (TIMS). We calculated and compared annual notification rates of different occupational groups and analyzed the epidemiological and clinical characteristics. The annual TB notification rates among general HCWs declined steadily from 2005 to 2011. On average, HCWs showed annual TB notification rates lower than the general population but higher than teachers. Recorded HCW TB patients averaged 35.5 years of age, with females outnumbering males (58.0% > 42.0%). The proportion of pulmonary tuberculosis (PTB) was higher among male than in the female patients (88.5% > 83.4%, *P* = 0.031). Our study suggested that general HCWs run a higher occupational risk than teachers although the two groups are socioeconomically comparable and that the priority should be given to the young female HCWs for TB prevention in healthcare institutions.

## 1. Introduction

Frequent exposure to *Mycobacterium tuberculosis* (MTB) puts healthcare workers (HCWs) at a high risk of infection with tuberculosis (TB) [1]. Compared with high-income countries, TB prevalence among health workers has been higher in low- and middle-income countries (LMICs), where the prevalence of latent tuberculosis infections (LTBI) has ranged from 33% to 79% [1,2,3,4]. China, with more than 1.3 million new cases in 2012, has the second largest TB epidemic [5]. To combat the spread of TB, China launched a national Tuberculosis Program (NTP) in 2001, which has effectively curbed the TB epidemic with a 60.9% drop in the prevalence rate of smear-positive TB from 2000 to 2010 [6]. Despite comprehensive control measures and impressive progress in the past decade, control measures for nosocomial infection were inadequate, as resources were mostly assigned preferentially to patient service [7]. A recent survey among 5235 HCWs reported that the annual prevalence rate of TB was above 600/100,000 in three provincial regions: Beijing, Inner Mongolia and Shanghai [8]. Prevalence of the multi-drug resistant tuberculosis (MDR-TB) has been found as high in China, which poses more challenges for TB control among HCWs [5].

The prevalence of TB and pulmonary tuberculosis (PTB) in the Chinese eastern provinces has been lower than in the central or west provinces [6]. Zhejiang, a southeast province with 11 prefectures and 90 counties, boasted an average *per capita* GDP of about $11,055 in 2013, ranking among the top five in China [9]. The province has a low TB prevalence. The reported prevalence rate of TB of the province was reported to be 68.86/100,000 in 2010, and was shown to be on a continuous decline [10]. However, TB prevalence among HCWs in the province has not been studied before, and baseline data remain very limited.

Introduced in China in 2005, the Tuberculosis Information Management System (TIMS) records each TB patient’s basic demographic information (sex, age, occupation, habitation, *etc*.), along with diagnosis and treatment [11]. It serves as a useful tool for monitoring TB patients. From 2005 to 2011, TIMS collected information on more than 280,000 TB patients in Zhejiang. We conducted a retrospective study focusing on the information of TB patients whose occupation was general HCW. The main objectives of this study were: (a) to understand baseline TB prevalence of general HCWs in the province; (b) to explore the epidemiological and clinical features of the HCWs with TB; (c) to provide advice on TB control among staff in health institutions.

## 2. Methods

### 2.1. Population and Data Source

The subjects for this study were general HCWs rather than specialty HCWs in TB departments. The information on all TB patients in Zhejiang from 2005 to 2011 was retrieved from the TIMS, and the information on HCWs and teachers with TB was extracted and analyzed. The teachers were chosen as the comparison group because the two groups have equivalent socioeconomic status and age structure [12]. The total numbers of HCWs and teachers each year were obtained from the Zhejiang Statistical Yearbook (2005–2011) [13]. The total number of HCWs in each prefecture from 2006 to 2011 was acquired from the website of Health and Family Planning Commission of Zhejiang Province (data for 2005 was absent) [14].

### 2.2. Data Analysis

The annual notification rates of TB and PTB (annual number of newly notified cases per 100,000 population) were calculated for the total population, HCWs and teachers and compared between each group. The TB notification rate was also calculated for HCWs in each prefecture. Based on the notification rates in different prefectures, a disease map was created to present the distribution of HCWs with TB in the province. A chi-square analysis was conducted to analyze the basic epidemiological characteristics, diagnosis and treatment of HCWs with TB.

### 2.3. Statistical Analysis

Microsoft Excel was used to organize the data derived from the TIMS. Statistical Package for Social Sciences (SPSS) version 19.0 by IBM (SPSS Inc, Chicago, IL, USA) was used for a single-factor chi-square test. Arc Gis 10.0 (ESRI, Redlands, CA, USA) was employed to present the geographical distribution of the disease.

### 2.4. Ethic Statement

The study was conducted in accordance with the Declaration of Helsinki, and the private information of the patients was not revealed. The study was approved by the Ethics Committee of Zhejiang Provincial Center for Disease Prevention and Control.

## 3. Results

### 3.1. Tuberculosis (TB) Pulmonary Tuberculosis (PTB) Notification Rates among Healthcare Workers (HCWs)

**Figure 1:**
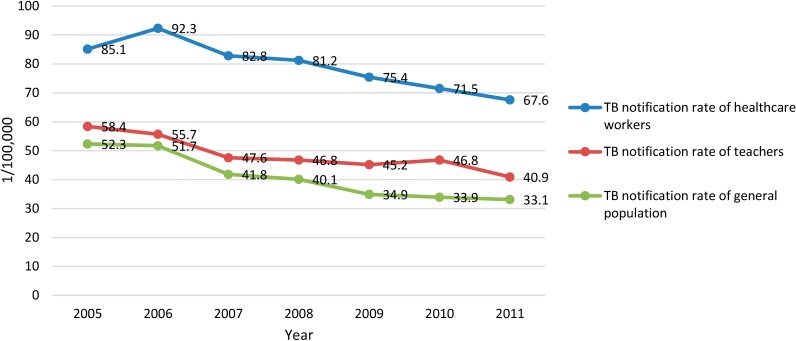
TB notification rate of the general population, HCWs, and teachers from 2005 to 2011 in Zhejiang Province, China (/100,000).

**Figure 2:**
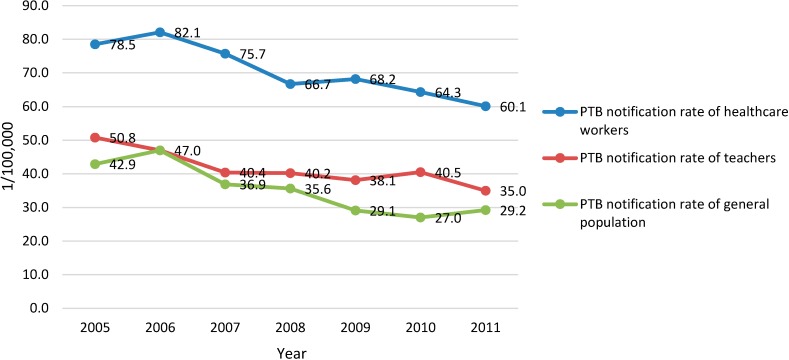
PTB notification rate of the general population, HCWs, and teachers from 2005 to 2011 in Zhejiang Province, China (/100,000).

In Zhejiang, the annual number of TB cases reported and recorded in the TIMS from 2005 to 2011 were 41,473, 45,208, 41,234, 41,087, 38,604, 37,037 and 36,792 in the general population; 138, 142, 131, 135, 140, 157 and 153 among the HCWs, and 206, 211, 172, 170, 151, 148 and 146 among the teachers. The annual TB notification rate of HCWs, showing a trend of decline, was lower than that of the general population with the differences between 24.7/100,000 and 36.6/100,000 but higher than that of the teachers with differences between 4.0/100,000 and 12.9/100,000 during the study period (Figure 1). The active PTB patients among the HCWs totaled 852, with 120, 120, 111, 116, 118, 136 and 131 for each year from 2005 to 2011, respectively. The annual numbers of PTB cases were 38,257, 40,212, 37,698, 33,750 34,918, 33,307 and 32,710 in the general population, and 169, 192, 152, 151, 126, 118 and 129 among the teachers. The annual PTB notification rate of the HCWs, also showing a trend of decline was lower than that of the general population and higher (except 2006) than that of teachers with the differences between 3.5/100,000 and 13.5/100,000 (Figure 2).

### 3.2. Demographic Characteristics

Among the reported HCWs with TB, 418 were male (42.0%) and 578 were female (58.0%). The average age of the TB patients was 35.5 years, with the youngest being 18 years, 493 (49.5%) under 30 years, 237 (23.8%) between 31 and 40 years, 103 (10.3%) between 41 and 50 years, 109 (10.9%) between 51 and 60 years, and 54 (5.4%) over 60 years. Those under 40 years of age numbered 730 (73.3%).

Among HCWs with active PTB, 370 (43.4%) were male and 482 (56.6%) were female. The average age of the PTB patients was 35.4 years, with 421(49.4%) under 30 years, 206(24.2%) between 31 and 40 years, 88(10.3%) between 41 and 50 years, 89(10.4%) between 51 and 60 years, and 48(5.6%) above 60 years, respectively. Younger HCWs (below 40) with PTB outnumbered the elder (over 40). (Table 1).

**Table 1:** The number of Tuberculosis (TB) patients among the Healthcare Workers (HCWs) in 11 prefectures of Zhejiang from 2006 to 2011 (number, annual notification rate: /100,000).

Cities	2006	2007	2008	2009	2010	2011
Hangzhou (HAZ)	34 (61.7 )	29 (47.3)	42 (65.5)	35 (51.2)	51 (68.9)	40 (49.7)
Huzhou (HUZ)	14 (101.7)	7 (47.9)	12 (74.8)	11 (62.3)	8 (43.2)	6 (30.8)
Jiaxing (JX)	7 (39.5)	14 (72.5)	10 (44.4)	8 (32.6)	9 (34.4)	7 (25.3)
Jinghua (JH)	12 (50.9)	9 (36.4)	7 (27.4)	14 (52.4)	14 (46.7)	9 (26.2)
Lishui (LS)	8 (71.6)	5 (42.9)	4 (33.6)	5 (40.0)	4 (28.4)	10 (63.5)
Ningbo (NB)	19 (50.1)	18 (43.4)	15 (34.6)	14 (29.9)	15 (29.8)	29 (52.3)
Quzhou (QZ)	10 (107.8)	8 (81.3)	6 (61.2)	7 (68.7)	13 (110.8)	8 (57.1)
Shaoxing (SX)	13 (62.8)	10 (47.0)	12 (53.9)	8 (33.0)	10 (38.4)	8 (27.4)
Taizhou (TZ)	13 (50.9)	17 (62.0)	9 (31.3)	9 (29.5)	13 (40.8)	16 (43.6)
Wenzhou (WZ)	9 (26.2)	10 (27.1)	15 (40.2)	25 (61.3)	17 (37.3)	19 (36.0)
Zhoushan (ZS)	3 (50.2)	4 (62.5)	3 (45.0)	4 (56.6)	3 (40.6)	1 (11.7)

### 3.3. Geographic Distribution

**Figure 3:**
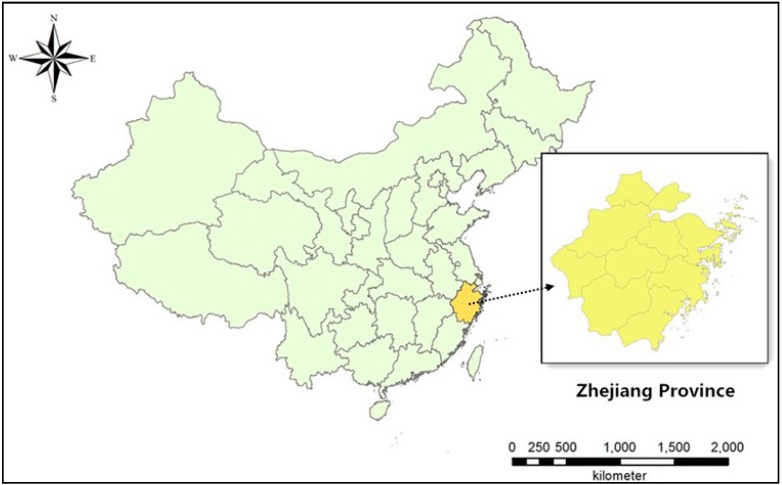
The location of Zhejiang in China.

**Figure 4:**
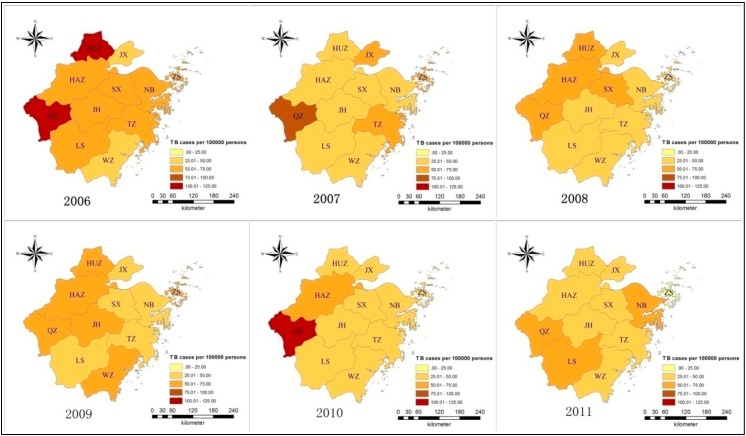
Tuberculosis (TB) notification rates of HCWs in 11 prefectures from 2006 to 2011 in Zhejiang (color depth indicating severity of epidemic at each 25/100,000 with the lightest level at 0–25/100,000 and the deepest at 100–125/100,000).

The TB notification rates of HCWs in the north prefectures (Huzhou, HUZ; Jiaxing, JX; and Shaoxing, SX) declined during the period from 2006 to 2011, but the rates fluctuated in the east and south prefectures (Ningbo, NB; Wenzhou, WZ; and Lishui, LS). Quzhou (QZ) in the west Zhejiang had a high epidemic (57.1/100,000–110.8/100,000) and Hangzhou (HAZ), the provincial capital, had a steady and moderate TB prevalence (47.3/100,000–68.9/100,000) (Table 1, Figure 3 and Figure 4).

### 3.4. Clinical Features of Tuberculosis (TB) among the Healthcare Workers (HCWs)

#### 3.4.1. Principal Diagnosis

In all the HCWs with TB, 852 (85.5%) patients were diagnosed with active PTB, 100 (10.0%) with tuberculous pleuritis and 44 (4.4%) with extrapulmonary TB. The proportion of PTB among the male HCWs was higher than among the female HCWs. The difference was statistically significant (*P* = 0.031). No difference was observed in the notification rates of PTB and other disease types between different age groups (Table 2).

**Table 2:** Healthcare Worker (HCW) Tuberculosis (TB) Patients with different principal diagnoses.

Factor	Pulmonary Tuberculosis		Tuberculous Pleuritis		Extrapulmonary Tuberculosis		Total
	N	%	N	%	N	%	
Gender ^*^							
Male	370	88.5	37	8.9	11	2.6	418
Female	482	83.4	63	10.9	32	5.5	578
Age ^**^							
−30	421	85.4	55	11.2	17	3.4	493
31–40	206	86.9	20	8.0	12	5.1	237
41–50	88	85.4	8	7.8	7	6.8	103
51–60	89	81.7	14	12.8	6	5.5	109
60-	48	88.9	4	7.4	2	3.7	54
Total	852	85.5	100	10.1	44	4.4	996

^*^ The *χ*^2^ –value is 6.96, *P*-value is 0.031, *P* < 0.05; ^**^ The *χ*^2^ –value is 6.60, *P*-value is 0.581, *P* > 0.05.

#### 3.4.2. Sputum Smear Tests

Sputum smear results were recorded in the 833 patients with pulmonary TB, of whom 231 turned out positive (27.7%) and 602 (72.3%) negative. No significant difference in the smear-positive rate was observed between male and female groups or between age groups (Table 3).

**Table 3:** Results sputum smear among Healthcare Worker (HCW) with Tuberculosis (TB).

Factor	Smear-Positive		Smear-Negative		*χ*^2^	*P*
	N	%	N	%		
**Gender**						
Male	96	26.6	265	73.4	0.41	0.521
Female	135	28.6	337	71.4		
**Age**						
≤30	108	26.0	308	74.0	4.78	0.310
31–40	52	26.4	145	73.6		
41–50	25	29.4	60	70.6		
51–60	27	31.0	60	69.0		
≥60	19	39.6	29	60.4		
Total	231	27.7	602	72.3		

#### 3.4.3. Treatment Outcomes

TIMS recorded the final clinical outcome of 661 patients with pulmonary TB, 193 from smear-positive patients and 468 from smear-negative patients. Among the former, 183 were cured with a cure rate of 94.8%. In the negative patients, 432 finished the course of treatment with a completion rate of 92.3%. The total treatment success (cure and treatment completion) rate was 91.7% among HCWs with PTB.

## 4. Discussion 

### 4.1. Tuberculosis (TB) Epidemic among General Healthcare Workers (HCWs)

This study showed that the notification rates of TB and PTB among general healthcare workers had declined from 2005 to 2011, which was consistent with those among the general population in Zhejiang province and China as a whole [6,10]. The population size of HCWs in Zhejiang has increased fast in recent years, which may contribute to the trend of decline evidenced in our study. The annual notification rates of TB and PTB among general HCWs in Zhejiang Province were lower than those of the total population but higher than those of teachers, whose socioeconomic status was comparable to HCWs [12].

Compared with other occupational groups, HCWs run a higher risk of TB infection during the process of diagnosis, treatment and delivery nursing care [15]. Meredith *et al*. found that the rates of notified TB cases in HCWs in England and Wales were 2–3 times those of other occupational groups with similar socioeconomic status [16]. A study in the Netherlands indicated that 28 out of 67 HCWs with TB were infected at work [2]. In countries with high TB epidemics, the situation is even worse [3,17,18,19,20]. The results of this study indicated that the notification rates of TB and PTB among general HCWs were higher than those among teachers in almost each year of the study period (except PTB rate in 2006), which might suggest a higher occupational risk of TB infection among HCWs in Zhejiang, China.

### 4.2. Epidemiological Characteristics of Tuberculosis (TB) among General Healthcare Workers (HCWs)

Among HCWs with TB and PTB, females outnumbered males, which was the opposite in the general population. A study conducted in England and Wales also claimed that female HCWs were more likely to develop TB than the general population [21]. The higher ratio of female TB patients among HCWs in this study may be attributable to the predominance of women in healthcare professions [22]. In this study, HCWs with TB averaged 35.5 years old, which is lower than other studies [22,23,24,25]. About half of the HCWs with TB were under 30 years of age, which is different from the disease distribution among the general population, where TB prevalence rises with the age [6]. The higher proportion of younger patients in Zhejiang Province might be explained by the younger age structure of its HCWs [26]. Moreover, young HCWs usually take on greater workloads, which could mean more opportunities to have contact with TB patients, increasing the risk of TB infection in healthcare settings. The overall notification rates of TB and PTB among the HCWs in most provincial prefectures were declining from 2005 to 2011. Some west prefectures (e.g., Quzhou) presented a different trend in TB rates, which could be due to an under-developed socioeconomic status and inadequate infection control measures.

### 4.3. Clinical Features of Tuberculosis (TB) among General Healthcare Workers (HCWs)

The proportion of smear-positive PTB patients was 27.7% among the general HCWs, which was lower than that of the general population (39.6%) in Zhejiang [10]. Among all the HCWs with TB, 85.5% were diagnosed with pulmonary TB, which was lower than that of the general population (90.0%) in 2010 [27]. The male patients had a higher proportion of pulmonary TB than the female patients. The treatment success rate of the HCWs with PTB (91.7%) was higher than that of all the PTB patients [10]. Better accessibility to medical resources and compliance to treatment schemes could be the reasons for the better outcomes for HCW patients [22].

### 4.4. Limitations

The fact that healthcare workers infected with TB is a sensitive issue for hospitals may result in omissions or concealment in reporting TB cases to TIMS. Therefore, the TB notification rate of HCWs may be underestimated. Due to the lack of demographic data on HCWs and teachers in the yearbook, the notification rates are unable to be standardized, which could limit the comparability of different groups. Moreover, for a number of factors, not all healthcare workers may have become infected in hospitals and the existing data could not be used to trace the source of infection. This warrants more field studies.

## 5. Conclusions

The TB notification rate among the HCWs in Zhejiang was lower than that of the general population, and presented a declining trend from 2005 to 2011. The notification rate of the HCWs was higher than that of the teachers, who had a similar socio-economic status to the HCWs. The result implied a higher occupational risk for TB infection among HCWs. The young female HCWs showed a higher notification rate for TB, and they should be the priority group for TB infection prevention in health institutions. The clinical features are similar among HCWs with pulmonary TB between genders and across ages. The results of this study warrants further field investigation for more information about the prevalence and risk factors of nosocomial TB among the HCWs in the province.
